# Reconstruction of Composite Stiffness Matrix with Array-Guided Wave-Based Genetic Algorithm

**DOI:** 10.3390/ma15248715

**Published:** 2022-12-07

**Authors:** Menglong Liu, Yaohui Zhang, Lun Li, Gongfa Chen, Fangsen Cui

**Affiliations:** 1 School of Mechanical Engineering and Automation, Harbin Institute of Technology, Shenzhen 518055, China; 2 School of Civil and Transportation Engineering, Guangdong University of Technology, Guangzhou 510006, China; 3 Institute of High Performance Computing, Agency for Science, Technology and Research, Singapore 138632, Singapore

**Keywords:** guided wave, carbon-fiber-reinforced polymer, genetic algorithm, stiffness matrix

## Abstract

Accurate measurement of the material parameters of composite in a nondestructive manner is of great significance for evaluating mechanical performance. This study proposes to use a genetic algorithm (GA) to reconstruct the stiffness matrix of carbon fiber reinforced polymer (CFRP) with array-guided wave (GW)-based GA. By comparing the numerically calculated GW dispersion curves with the experimental wave number-frequency contour calculated with a two-dimensional Fourier transform (2D-FFT), the matching coefficient is directly obtained as the objective function of the GA, avoiding the overhead of sorting out the respective GW modes. Then the measured stiffness matrix with tensile testing and the longitudinal wave in the unidirectional CFRP is compared with the reconstructed parameters from unidirectional, cross-ply, and quasi-isotropic CFRPs with the GA. For the four independent parameters, excluding $C_{12}$, an average value of 11.62% for the maximum deviation is achieved among the CFRPs with three stacking sequences, and an average deviation of 11.03% in unidirectional CFRPs is achieved for the parameters measured with different methods. A further correction of fiber orientation results in a relative deviation of only 2.72% for the elastic modulus along the tensile direction, and an expansion of the GW frequency range for the GA narrows down the relative deviation of $C_{12}$ to 3.9%. The proposed GW-based GA opens up a way of in situ and nondestructive measurement for the composite stiffness matrix.

## 1. Introduction

With the recent improvement in manufacturing technology, a variety of materials are woven, wound, laminated, etc., to form new composite materials, such as fiber-reinforced polymer laminates, Kevlar, ceramic matrix composite materials, etc. [1,2,3]. Compared with traditional metal materials, these new composite materials have significantly different structural forms and material properties, which will directly affect the mechanical properties and intended functions of the material. Moreover, in the processing, manufacturing, and service period of the materials, the molding process conditions (temperature, pressure, time, casting, forging, etc.), external loads, material aging, environmental temperature, humidity changes, etc. will inevitably lead to changes in the mechanical parameters of the material, or cause a change in the size, shape, and performance of the structure. For example, the elastic modulus value of fiber-reinforced composites can be used to characterize (1) whether the material meets the manufacturing requirements and (2) the real-time performance during service [4]. To withstand high temperatures, the surface layer of ceramic matrix composites will erode during service [5]. The wall thickness of the pipeline structure is often used as an important evaluation criterion for the severity of pipeline corrosion [6]. If the changes in the above-mentioned structural thickness and material parameters cannot be evaluated timely and accurately, they will seriously affect the normal service or functioning of the structure, thereby causing damage to the structure. Therefore, for these complex materials and structures, it is necessary to accurately characterize the structural and material parameters [7].

There are four main existing experimental techniques for measuring material parameters (mainly elastic modulus or stiffness matrix), including techniques based on quasi-static loading, sub-resonance, resonance, and wave propagation. The quasi-static-loading-based technology uses an external loading device to load a specimen with a specific size, and a single test can only obtain a single material parameter [8,9]. The sub-resonance-based technique calculates the elastic modulus and internal friction according to the acquired load-displacement response relationship when the excitation frequency of the structure is less than the first-order resonance frequency. This method is suitable for materials under laboratory conditions. Representative equipment includes commercial dynamic mechanical analyzers and Kê pendulum clocks [10]. Resonance technology is currently the most widely used method for testing material parameters in the laboratory. Typical methods include the free-free beam method [11] and the pulse excitation method [12], both of which are based on the bending or torsional resonance frequency of a rectangular bar specimen to measure the material’s elastic modulus. However, the accuracy of this method is strongly dependent on the support location and support method. The resonance ultrasonic spectroscopy method [13], which is also based on resonance technology, obtains multiple resonance frequencies through a single measurement and then adopts the inverse algorithm to match the resonance frequencies measured by numerical calculation and experiment and can measure all 21 independent elastic constants with a sample of specific dimensions under laboratory conditions.

From the review of the four main existing experimental techniques for measuring material parameters, it is concluded that these techniques mainly focus on the characterization of material parameters in the laboratory with samples of specific sizes. Thus, there still exists a gap in in situ and nondestructive measurements. Instead, stress waves exhibit the characterization potential of material and structural parameters, which control the characteristics of wave propagation. Several bulk wave-based techniques were developed to obtain the material parameters [14]. For composites, there exist multiple independent elastic modulus parameters whose parameter identification was made possible with bulk wave data propagating in multiple directions [15,16,17]. In addition, multiple material parameters jointly determine the wave velocity. It is necessary to calculate the error between the multiple measurement data and the theoretical value, establish the objective function (OF), and use the GA [18], Newton-Raphson method [19], simplex method [18], and other inverse problem algorithms to obtain the optimal solution for multiple parameters. The bulk-wave-based technique is usually used with thick materials, as a thin sample will cause a large measurement error in wave velocity.

Different from the propagation of ultrasonic bulk waves, Guided waves (GWs) propagate along the guided direction of the waveguide and feature a propagation velocity that varies with the frequency and the coexistence of multiple modes [20,21]. Thus, the propagation characteristics are jointly governed by the geometrical dimensions of the waveguide structure and the material’s mechanical parameters. To obtain the GW dispersion curve, the commonly used signal excitation and acquisition methods include the piezoelectric wafer [22], non-contact laser [23,24], ultrasonic piezoelectric array probe [25], etc. Compared with the contact-based signal acquisition method that the probe may influence the signal quality, and the non-contact method offers more accurate data.

In the research on constructing a matching index between the theoretical and experimental GW characteristics, the multi-mode and dispersion characteristics of GWs bring rich information but also pose challenges. Foiret et al. [25] used a linear array to collect the GW signal on a cortical bone, constructed the OF using the difference between the theoretical calculation and the experimental results, and obtained the thickness and wave speed of the bone. The GWs can only be extracted manually by trained researchers, making the method difficult to implement practically. Eremin et al. [26] manually picked the fundamental $A_{0}$ and $S_{0}$ modes, whose wavelengths were input into a GA for parameter reconstruction of unidirectional and cross-ply carbon fiber reinforced polymer (CFRP) laminate. Further, an unsupervised learning method based on the mode separation of a GW was proposed to characterize the material parameters of multiple isotropic materials [27]. Similarly, Okumura et al. [28] adopted a diagonal loading technique to quickly extract a GW curve in an isotropic material. Lu et al. [29] proposed a hybrid particle swarm-based-simulated annealing optimization technique to obtain the elastic properties of the isotropic plate structure, but multiple GW curves need to be extracted first, which is difficult to apply in complex structures. Zhao et al. [23] first converted the experimentally measured group velocities of a GW fundamental mode in the 360-degree circumferential directions into phase velocities, manually selected a specific mode, and then used the error between the theoretical and experimental phase velocities to construct the OF. Hu et al. [30] further developed the hybrid Lasso regression to improve the experimental signal-to-noise ratio of a GW along one single direction for the reconstruction of the material stiffness matrix.

In the above research, the dispersion curve of the GW needs to be extracted from the experimentally acquired data either manually or with some signal processing methods, which may be quite complex when higher-order GW modes are involved in a wide frequency range. In order to overcome the dependence on the extraction of GW modes, Bochud et al. [31] proposed a positive and negative value method by substituting the experimental contour with a wavenumber-frequency domain into the theoretical GW equation for single-layer weakly anisotropic materials, which can automatically construct an OF, but the method is limited to single-layer materials and does not demonstrate the ability to generalize to complex multilayer structures with strong anisotropy such as CFRP.

In summary, the existing structural and material parameter reconstruction methods based on ultrasonic bulk waves are mainly used for large-thickness materials. Meanwhile, the existing techniques based on a GW, because the multi-mode and dispersion characteristics make it difficult to effectively identify each mode, rely heavily on the manual classification of GW modes from experimental results to establish a matching target that minimizes experimental and computational errors. In addition, the information from different GW propagation directions has yet to be fully used for a more robust parameter reconstruction of a CFRP with strong anisotropy.

Addressing the above problems, this study proposes an automatic reconstruction of the CFRP material stiffness matrix with the GW-based GA. The stiffness matrix for comparison in this study is obtained based on data from the tensile loading and longitudinal wave. In addition, the proposed method does not rely on any manual selection of specific GW modes and adopts a GA to realize the reconstruction of the stiffness matrix of lamina based on a GW in CFRP laminates with unidirectional (UD), cross-ply (CP), and quasi-isotropic (QI) stacking sequences.

The rest of the paper is organized as follows. Section 2 details the experimental setups and methods of static tensile loading and longitudinal wave testing to obtain one set of the stiffness matrix and the GW testing to obtain multiple sets of the stiffness matrix of CFRPs with different stacking sequences. Section 3 analyzes the sensitivity of material parameters on GW characteristics, the deviation of reconstructed parameters, and the further refinement of several key parameters. Concluding remarks are provided in Section 4.

## 2. Materials and Methods

The static tensile loading and longitudinal wave testing were performed first to obtain the stiffness matrix of UD; then, the GW testing was performed to acquire the array of GW signals, which were input into the GA for the reconstruction of the stiffness matrix.

### 2.1. Experimental Setup

#### 2.1.1. Static Tensile Loading

For the unidirectional CFRP sample with the fiber along the *x* axis, *y*-*z* plane can be treated approximately as a plane of isotropy, and thus the stress (σ) to strain (ε) relation is given as

(1) $$\left[\begin{array}{c} \sigma_{1}\\ \sigma_{2}\\ \sigma_{3}\\ \sigma_{4}\\ \sigma_{5}\\ \sigma_{6} \end{array}\right]=\left[\begin{array}{cccccc} C_{11} & C_{12} & C_{12} & 0 & 0 & 0\\ C_{12} & C_{22}{}^{†} & C_{23} & 0 & 0 & 0\\ C_{12} & C_{23} & C_{22}{}^{†} & 0 & 0 & 0\\ 0 & 0 & 0 & C_{44} & 0 & 0\\ 0 & 0 & 0 & 0 & C_{55} & 0\\ 0 & 0 & 0 & 0 & 0 & C_{55} \end{array}\right]\left[\begin{array}{c} \varepsilon_{1}\\ \varepsilon_{2}\\ \varepsilon_{3}\\ \varepsilon_{4}\\ \varepsilon_{5}\\ \varepsilon_{6} \end{array}\right],$$

where $\sigma_{i}$ denotes the normal or shear stress, $\varepsilon_{i}$ denotes the normal or engineering shear strain, $C_{ij}$ denotes the parameters of stiffness matrix, in which $C_{22}{}^{†}$ is not independent and can be given by the following linear combination of two other elements

(2) $$C_{22}{}^{†}=C_{23}+2C_{44},$$

leaving the number of independent elements in the stiffness matrix of Equation (1) to be five. These five independent elements can also be converted into five numbers consisting of elastic moduli $E_{1}$, $E_{3}$, and $G_{13}$, and Poisson’s ratios $v_{13}$ and $v_{23}$.

**Figure 1:**
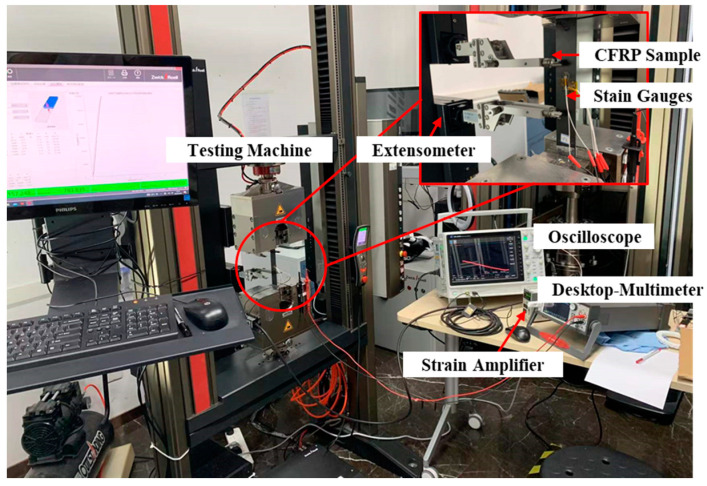
Experimental setup of static loading.

Static tensile loading of the UD sample, as displayed in Figure 1, was performed to measure four parameters, including $E_{1}$, $E_{3}$, $v_{13}$, and $G_{13}$. The parameters of CFRP samples, load, and sensor for tensile loading are listed in Table 1. Every third sample of the nine samples (250 mm × 25 mm) was cut along the x direction, 45° to the x direction, and along the z direction, respectively. A universal testing system (ZwickRoell^®^ 20 kN Allround tabletop, Ulm, Germany) was used for tensile loading. A 350 Ω strain gauge (BE350-3AA-P100, ZEMIC Group, Xi’an, China) bonded normal to the loading direction was connected to the dynamic strain amplifier (KYOWA^®^ DPM-911B, Tokyo, Japan), whose output voltage was acquired with an oscilloscope (Keysight^®^ Infiniium MXR058A, Colorado Springs, CO, USA). Another 350 Ω strain gauge bonded along the loading direction was connected directly to the digital multimeter (Keithley^®^ DMM7510, Tektronix, Inc., Beaverton, OR, USA), whose acquired value of electrical resistance was proportional to the measured strain value.

#### 2.1.2. Longitudinal Wave Testing

**Figure 2:**
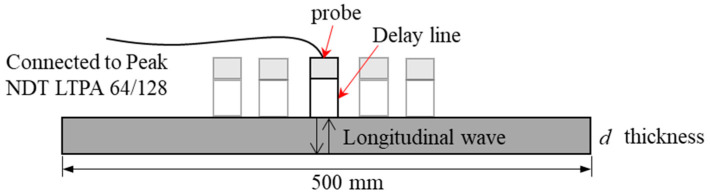
Experimental setup of longitudinal wave testing.

To measure the last parameter $v_{23}$ in the UD sample, longitudinal wave testing was performed, as displayed in Figure 2. A delay line piezoelectric probe with a central frequency of 15 MHz was excited via the conventional channel of the multi-channel pulse exciter/receiver (Peak NDT^®^ LTPA 64/128, Derby, UK), acting as both pulse transmitter and receiver unit. Five random locations were selected to calculate the average arrival time of echo signals. A sampling rate of 100 MHz was adopted for signal acquisition.

#### 2.1.3. Guided Wave Testing

Laminates (500 mm × 500 mm) with three stacking sequences were fabricated, which are listed in Table 2, together with the average thickness and density.

**Figure 3:**
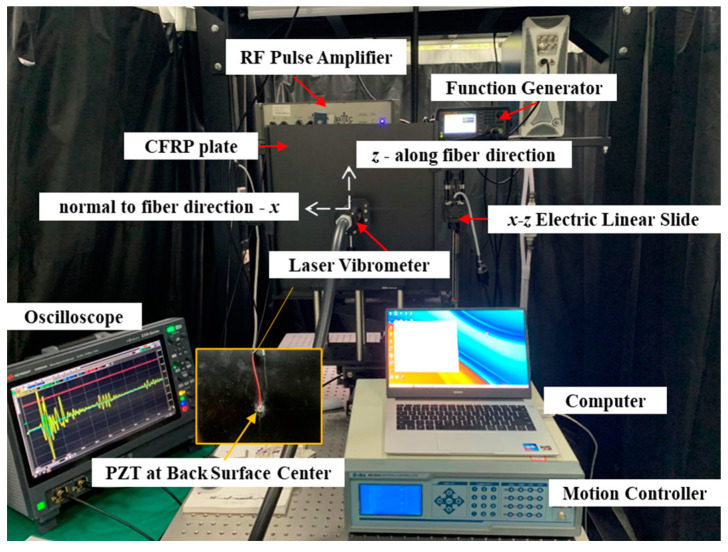
Experimental setup of GW testing.

The GW testing was performed as shown in Figure 3, with key parameters listed in Table 3. A three-cycle Hanning window modulated sinusoidal signal at a center frequency of 400 kHz was amplified to 800 Vpp via the gated high-power radio frequency pulse amplifier (Ritec^®^ GA-2500A, Warwick, RI, USA), and output to the piezoelectric wafer (Φ 8 mm, 0.48 mm thick) bonded on the surface of the CFRP sample. The wave signal was acquired with the laser vibrometer (Tecnar^®^ LUS discovery, Saint-Bruno, QC, Canada) after a sample averaging 512 times. The plate was fixed to a stepper motor with a movement increment of 1 mm and a total movement distance of 128 mm. In this way, out-of-plane displacements at 128 points were acquired along both the horizontal (x) and vertical (z) directions.

### 2.2. Methods

#### 2.2.1. Derivation of Material Parameters with Tensile Loading

The derivation of the four parameters based on the static tensile loading of UD is as follows:

(1) Young’s modulus along the fiber direction $E_{1}$;
  (3) $$E_{1}=\frac{\sigma_{1-1}}{\varepsilon_{1-1}}=\frac{N_{1}}{A\varepsilon_{1-1}}$$
  where $\sigma_{1-1}$, $\varepsilon_{1-1}$, and $N_{1}$ denote the longitudinal stress, strain, and loading force with the sample cut along the fiber direction, and A is the cross-sectional area.
(2) Young’s modulus normal to the fiber direction $E_{3}$;
  (4) $$E_{3}=\frac{\sigma_{3-3}}{\varepsilon_{3-3}}=\frac{N_{3}}{A\varepsilon_{3-3}}$$
  where $\sigma_{3-3}$, $\varepsilon_{3-3}$, and $N_{3}$ denote the longitudinal stress, strain, and loading force with the sample cut normal to the fiber direction.
(3) Poisson’s ratio $v_{13}$;
  (5) $$v_{13}=\frac{\varepsilon_{3-1}}{\varepsilon_{1-1}}$$
  where $\varepsilon_{3-1}$ and $\varepsilon_{1-1}$ denote the transverse and longitudinal strains with loading to the sample along the fiber direction.
(4) Shear modulus $G_{13}$;
  The Young’s modulus $E_{45^{\circ }}$ of the sample cut along 45° to the x direction is related to $E_{1}$, $E_{3}$, $v_{13}$, and $G_{13}$ as follows:
  (6) $$\frac{1}{E_{45^{\circ }}}=\frac{1}{4}(\frac{1}{E_{1}}+\frac{1}{E_{3}}+\frac{1}{G_{13}}-\frac{2v_{13}}{E_{1}})$$
  thus $G_{13}$ can be calculated provided the known $E_{1}$, $E_{3}$, $v_{13}$, and $E_{45^{\circ }}$.

#### 2.2.2. Derivation of Material Parameter with Longitudinal Wave

1. Poisson’s ratio $v_{23}$;
  The longitudinal wave testing was performed to measure the last parameter $v_{23}$, as illustrated in Figure 2. The average velocity of the longitudinal wave is measured experimentally as
  (7) $$v_{L}=\frac{1}{5}\sum_{i=1}^{5}\frac{2d_{i}}{\Delta t_{i}}$$
  where d is the measured thickness and Δt is the time of flight of the pulse-echo. The measurement is performed at five random locations with i=1, 2, 3, 4, 5. The velocity is related to $E_{3}$ and $v_{23}$ as
  (8) $$v_{L}^{2}\rho =C_{22}=\frac{1-v_{12}v_{21}}{N}E_{2}$$
  where
  (9) $$N=1-2v_{12}v_{21}-v_{23}v_{32}-2v_{21}v_{32}v_{13}$$
  via which $v_{23}$ can be derived.

#### 2.2.3. Derivation of Material Parameters with GW-Based GA

**Figure 4:**
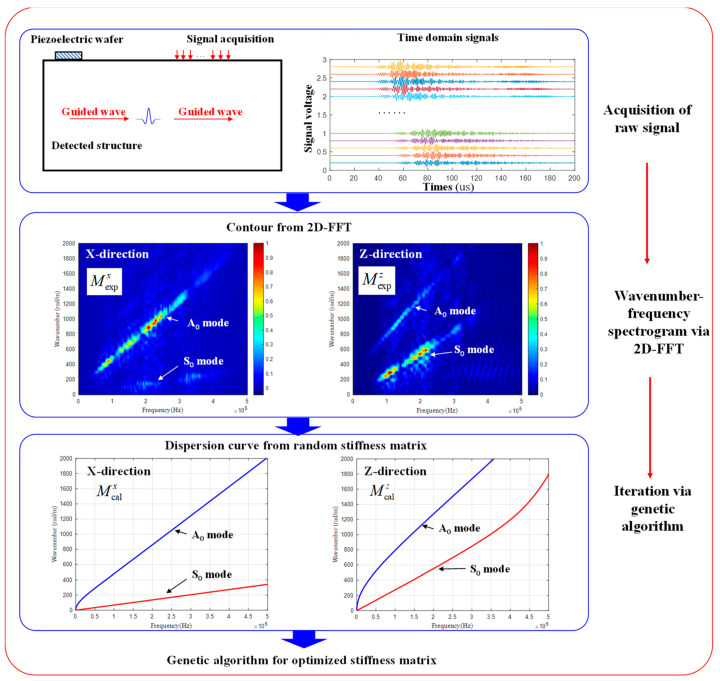
Flowchart of parameter reconstruction based on GA.

The flowchart of parameter reconstruction with GW-based GA is illustrated in Figure 4. Based on the desired image resolution in the frequency domain, the 128 time-domain signals along both directions acquired with the laser vibrometer were zero-filled to desired lengths before the operation of 2D-FFT. Then the original wave number at [1000/128 × 2π: 1000/128 × 2π: 2000π] was linearly interpolated to the desired resolution. Finally, the experimental spectrogram M_exp_ of frequency-wave number domain at the range of 0–500 kHz and 0–2000 rad/m with the desired resolution was generated. On the other hand, the theoretical dispersion curve of GW was initially calculated with a semi-analytical finite element (SAFE) developed by the authors [32], in which the wave number was swept from 0 to 2000 rad/m at the desired resolution to obtain the exact solution of the frequency with the input material parameters. Then the exact solution was rounded to the nearest frequency value at the desired resolution in the frequency domain to obtain the theoretically calculated image $M_{\mathrm{cal}}$.

In order to reconstruct $C_{11},C_{12},C_{22},C_{23},{\mathrm{and}\;C}_{66}$ simultaneously, both the signals along the 0° (x) and 90° direction (z) were used in the OF described as

(10) $$F(C_{11},C_{12},C_{22},C_{23},C_{66})=M_{\mathrm{cal}}^{x}M_{\exp}^{x}+M_{\mathrm{cal}}^{z}M_{\exp}^{z}$$

For the UD for which some parameters can be decoupled with the GW along the 0° (x) and 90° direction (z), which is analyzed in Section 3.1, the OF is described as

(11) $$\{\begin{array}{c} F(C_{22},C_{23})=M_{\mathrm{cal}}^{z}M_{\exp}^{z}\\ F(C_{11},C_{12},C_{66})=M_{\mathrm{cal}}^{x}M_{\exp}^{x} \end{array}$$

**Figure 5:**
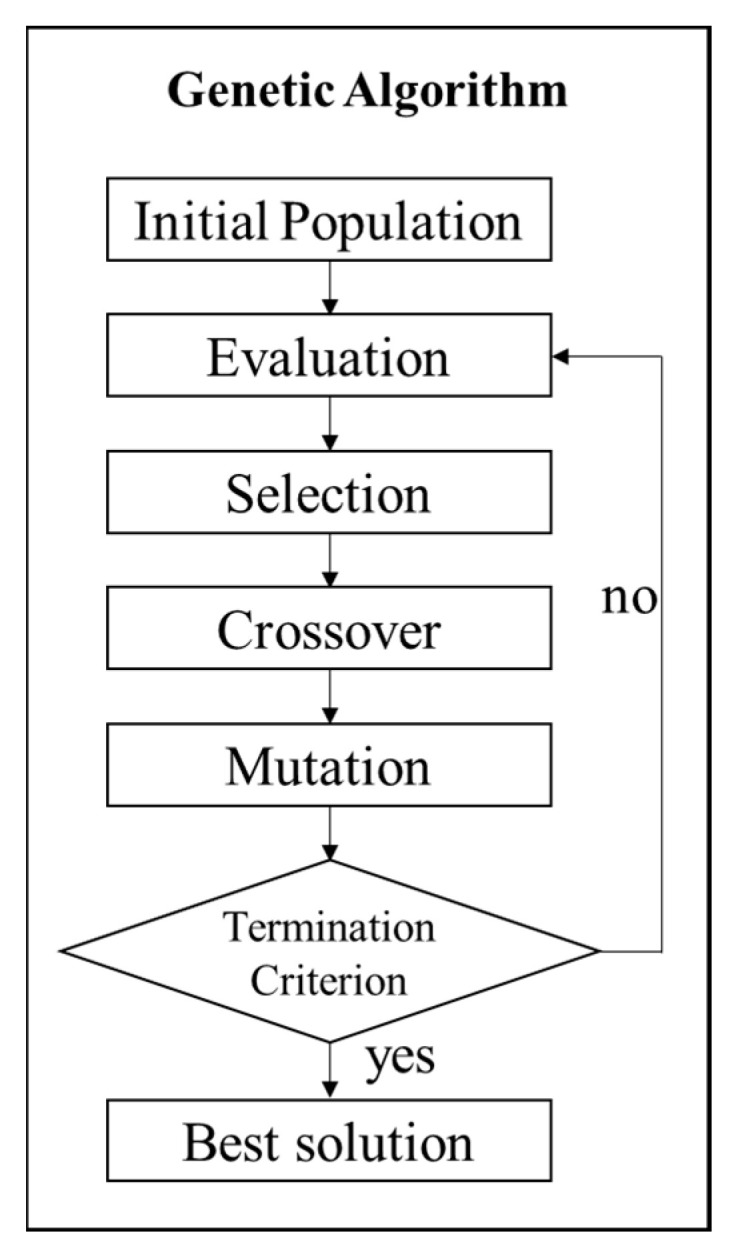
Flowchart of GA for inverse reconstruction of stiffness matrix.

Figure 5 shows the flowchart of GA for the reconstruction of the stiffness matrix to seek the optimum parameter with the maximum value of OF. A computer with a CPU of Intel(R) Core(TM) i7-9700 CPU@3.00GHz and RAM of 32 GB (Intel Corporation, Santa Clara, CA, USA) was used. MATLAB was adopted to fulfill the calculation of both the GW dispersion curve and GA. The algorithm started randomly generating an initial population of 64. The upper and lower bounds for each parameter were set as 150% and 50%, respectively, of the parameter derived from the static tensile loading and longitudinal wave testing. Every initial parameter increment was set as 0.1 GPa. A uniform crossover with a probability of 0.9 and mutation operations with a probability of 0.5 were then performed. Individual fitness was evaluated by the OF defined in Equations (10) and (11). The algorithm was considered converged when the first 16 values of each parameter with the largest OF remained unchanged.

## 3. Results

According to the five calculated material parameters based on the static tensile loading and longitudinal wave testing, the sensitivity of each parameter on the GW dispersion curve is analyzed, followed by the reconstruction of the material parameter with a GW-based GA.

### 3.1. Sensitivity Analysis of Parameter on GW Dispersion Curve

According to the results from the static tensile loading and longitudinal wave testing, all five parameters are listed in Table 4, together with the calculated stiffness matrix.

**Figure 6:**
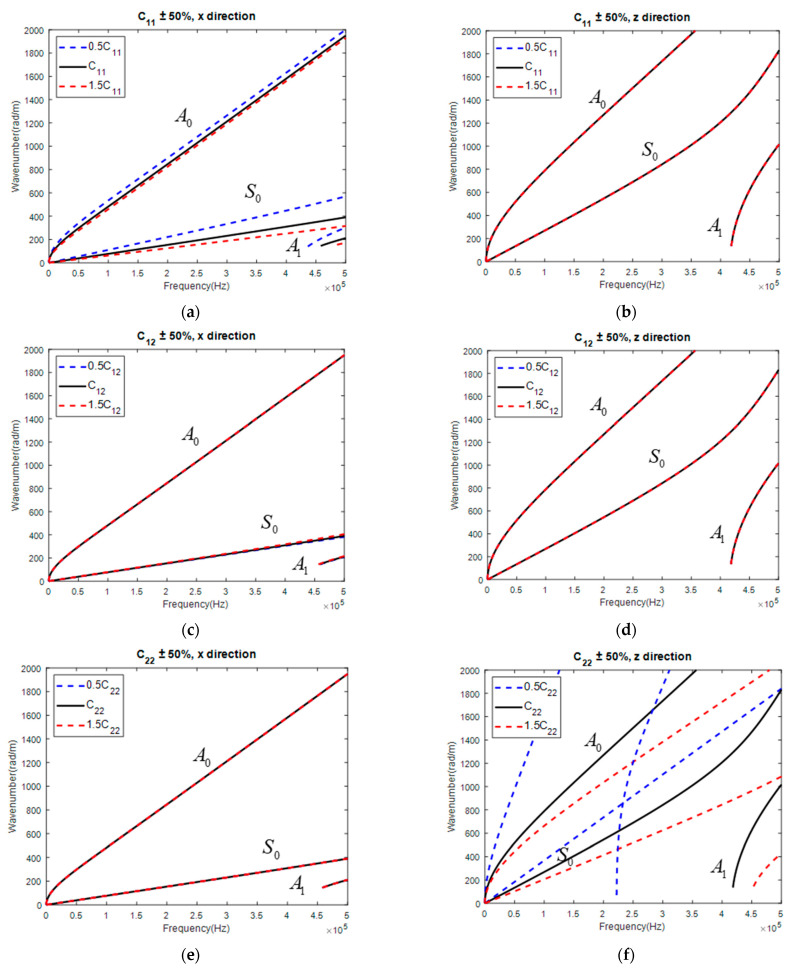

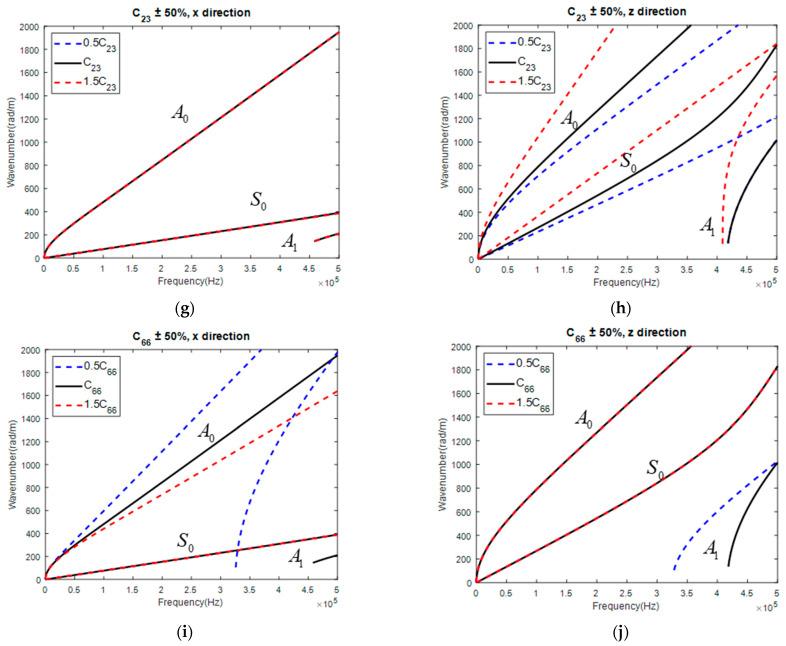
Influence of parameter on GW dispersion curve in UD: $C_{11}$ on GW along (**a**) x and (**b**) z direction; $C_{12}$ on GW along (**c**) x, and (**d**) z direction; $C_{22}$ on GW along (**e**) x and (**f**) z direction; $C_{23}$ on GW along (**g**) x and (**h**) z direction; $C_{66}$ on GW along (**i**) x and (**j**) z direction.

Taking the UD for investigation, each parameter was changed by 50% while keeping other parameters unchanged, whose corresponding GW dispersion curves are displayed in Figure 6. It is concluded that

(1) $C_{11}$ mainly influenced $S_{0}$ along the fiber direction while independent from the GW normal to the fiber direction.
(2) $C_{12}$ slightly influenced the GW along the fiber direction while independent from that normal to the fiber direction.
(3) $C_{22}$ was almost independent of the GW along the fiber direction while largely influencing the GW normal to the fiber direction.
(4) $C_{23}$ slightly influenced the GW along the fiber direction while largely influencing the GW normal to the fiber direction.
(5) $C_{66}$ largely influenced the GW along the fiber direction while independent from the GW normal to the fiber direction.

Thus, while using the GW for the reconstruction of the stiffness matrix of UD, $C_{12}$ might exhibit a large error, as a GW along both directions was hardly sensitive to its change. In addition, considering that only $C_{22}$ and $C_{23}$ determined the GW normal to the fiber direction, the five independent parameters were decoupled into two groups. The 1st group included $C_{22}$ and $C_{23}$, whose values were reconstructed with a GW normal to the fiber direction. Then, the obtained $C_{22}$ and $C_{23}$ were input into the GA as known parameters to reconstruct the value of the 2nd group of parameters including $C_{11}$, $C_{12}$, and $C_{66}$, with a GW along the fiber direction.

**Figure 7:**
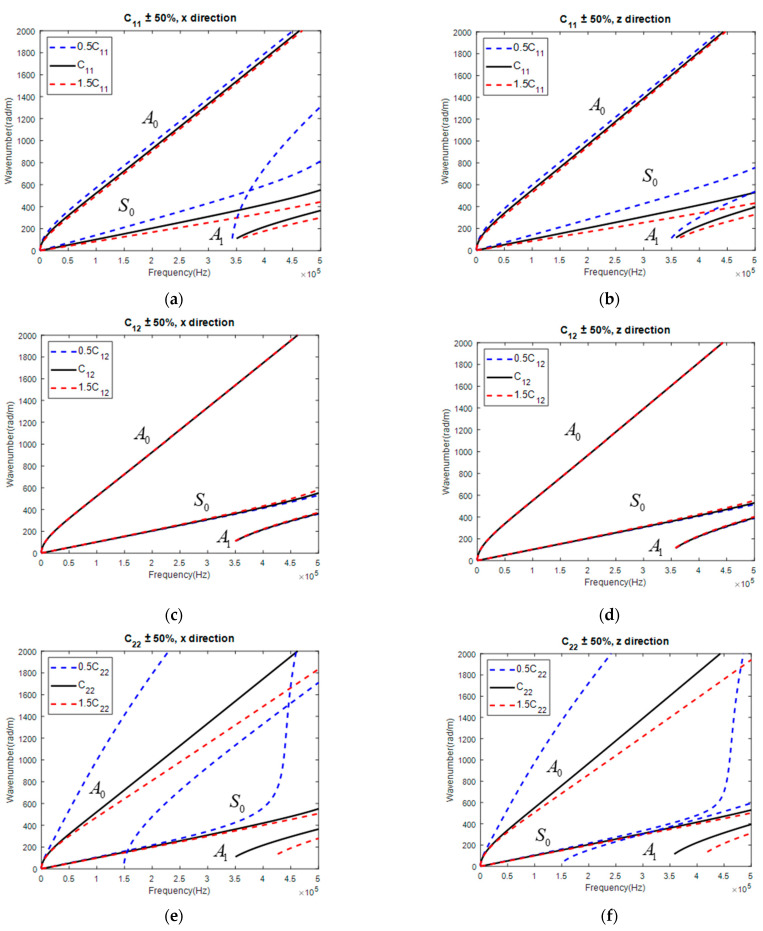

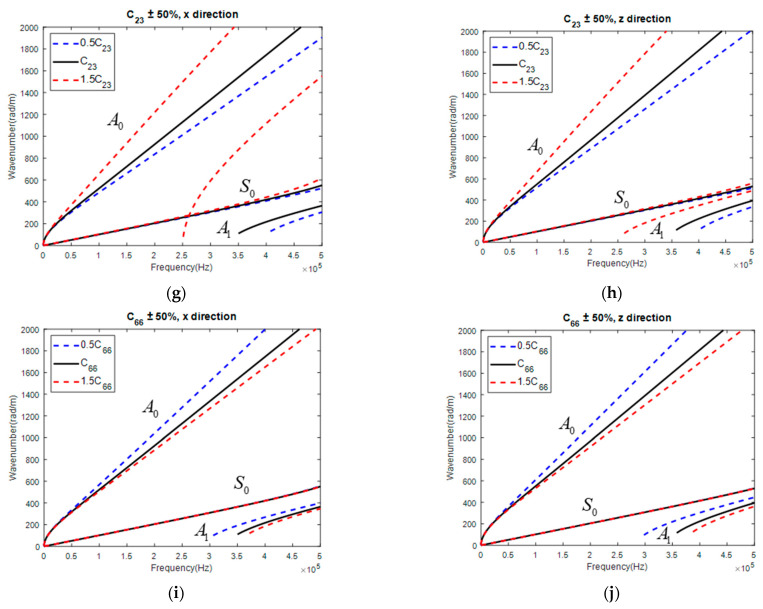
Influence of parameter on GW dispersion curve in CP: $C_{11}$ on GW along (**a**) x and (**b**) z direction; $C_{12}$ on GW along (**c**) x and (**d**) z direction; $C_{22}$ on GW along (**e**) x and (**f**) z direction; $C_{23}$ on GW along (**g**) x and (**h**) z direction; $C_{66}$ on GW along (**i**) x and (**j**) z direction.

**Figure 8:**
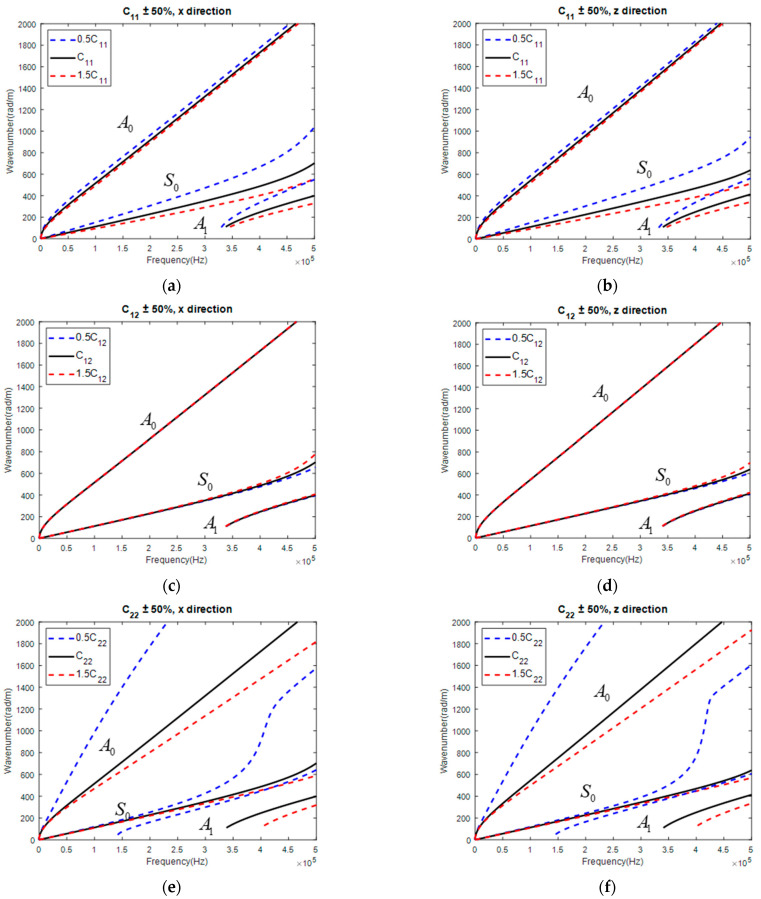

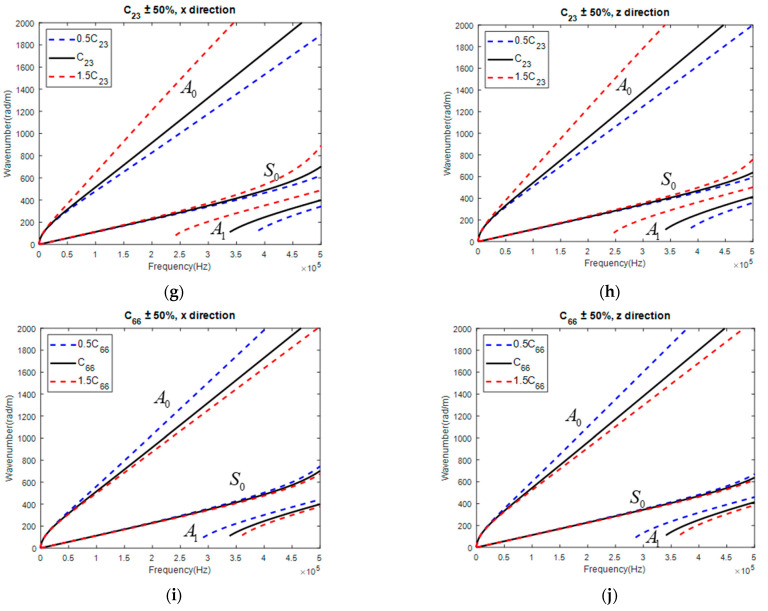
Influence of parameter on GW dispersion curve in QI: $C_{11}$ on GW along (**a**) x and (**b**) z direction; $C_{12}$ on GW along (**c**) x and (**d**) z direction; $C_{22}$ on GW along (**e**) x and (**f**) z direction; $C_{23}$ on GW along (**g**) x and (**h**) z direction; $C_{66}$ on GW along (**i**) x and (**j**) z direction.

Taking the CP and QI for further investigation, the corresponding GW dispersion curves are displayed in Figure 7 and Figure 8, respectively. It was concluded that $C_{12}$ was still insensitive to GW along both directions, which made it hard to be correctly identified. Except for that, other parameters showed the potential of being correctly reconstructed, as the change of parameters all influenced the dispersion curve of the GW in both directions. Thus, all five independent parameters were simultaneously reconstructed with a GW along both directions.

### 3.2. Reconstruction of Stiffness Matrix of CFRP with GW-Based GA

With the proposed GW-based GA, the reconstructed values of the stiffness matrix with different image resolutions (251 × 251, 501 × 501, 1001 × 1001, and 2001 × 2001) and the maximum relative deviation in percentage defined below are listed in Table 5,

(12) $$\delta_{ij}^{k}=\left|\frac{C_{ij}^{k}-{\bar{C}}_{ij}^{k}}{{\bar{C}}_{ij}^{k}}\times 100\right|$$

where k=1, 2, and 3 denote UD, CP, and QI, respectively, and ${\bar{C}}_{ij}^{k}$ denotes the mean value of $C_{ij}^{k}$. The mean values of the maximum deviation in percentage are 27.46%, 12.40%, 17.35%, and 11.71% for image resolutions 251 × 251, 501 × 501, 1001 × 1001, and 2001 × 2001, respectively. Hence, the accuracy of the parameter reconstruction is well guaranteed at the image resolution of 501 × 501, which is adopted for further analysis. Note that the calculation time before convergence is largely dependent on the random initial values and has an average time of around 20 min.

It is concluded from the reconstruction result with the image resolution of 501 × 501 that

(1) The maximum deviation percentage of $C_{11}$ is only 1.42%, which indicates that $C_{11}$ reconstructed from the GW-based GA is highly accurate and robust.
(2) $C_{12}$ exerts negligible influence on the GW dispersion curves along both the x and z directions for CFRPs with the investigated three stacking sequences. Thus, a large maximum deviation of up to 17.5% should be expected.
(3) The maximum deviation percentage of $C_{22}$ is only 7.80%, which indicates that $C_{22}$ reconstructed from the GW-based GA is highly accurate and robust. The mean value of the three reconstructed values of $C_{22}$ with CFRPs of three different stacking sequences is calculated as 10.20 GPa, which is extremely close to the value of 10.28 GPa derived with tensile loading and longitudinal wave. As illustrated in Figure 6f, $C_{22}$ exerts a significant influence on the GW dispersion curve normal to the fiber direction in unidirectional CFRP. Thus, the reconstructed value of $C_{22}$ can be highly trusted.
(4) $C_{23}$ shows a maximum deviation of 23.63%. The reconstructed values from CP and QI are smaller than that from UD, which may be partially attributed to the lower sensitivity of a GW in CP and QI to $C_{23}$ than that in UD.
(5) $C_{66}$ shows a maximum deviation of 13.63%. As $C_{66}$ exerts an influence on a GW along the fiber direction (see Figure 6i) in UD and along both directions in CP (see Figure 7i,j) and QI (see Figure 8i,j), the derived result should be trusted.

**Figure 9:**
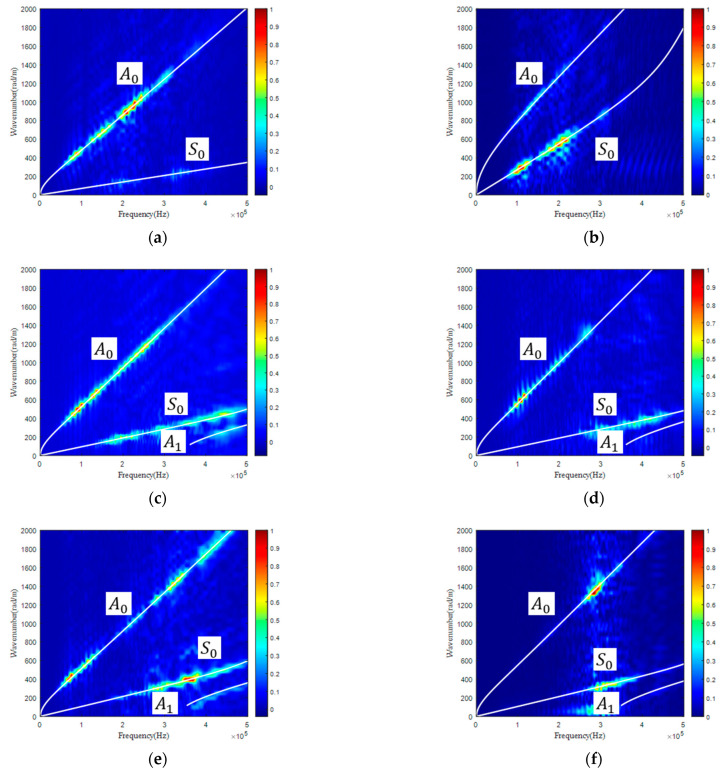
Experimentally obtained frequency and wave number contour superposed with the GW dispersion curve calculated with the reconstructed stiffness matrix: UD along (**a**) x direction and (**b**) z direction; CP along (**c**) x direction and (**d**) z direction; QI along (**e**) x direction and (**f**) z direction.

Based on the reconstructed parameters, the dispersion curves were calculated and superposed with the experimentally obtained GW frequency and wave number contour, as displayed in Figure 9. The magnitude of acquired GW significantly varied among modes, frequencies, and laminates with specific stacking sequences. E.g., the $S_{0}$ mode along the fiber direction of UD was hardly visible (see Figure 9a), higher modes along the x direction were clearly acquired in CP and QI, and both $A_{0}$ and $S_{0}$ modes were acquired only around the frequency range [100 kHz, 250 kHz]. Despite partially missing information and the existence of measurement noise, all the contours were well matched with the GW dispersions curves, which proved the correctness of the adopted GA.

As $C_{11}$, $C_{12}$, and $C_{66}$ mainly influence the GW along the fiber direction in UD, and $C_{22}$ and $C_{23}$ jointly decide the GW normal to the fiber direction, the parameter reconstruction for UD can be decoupled into two groups. By contrast, for CP and QI, all five parameters are coupled together to jointly determine the GW propagation along either direction. Thus, it is assumed that the parameters reconstructed from UD may be more accurate than those from other samples. In addition, the tensile loading and the longitudinal wave test are performed on UD to derive another set of parameters. Considering the mentioned two aspects, the parameters reconstructed from the GW in UD are used as a benchmark to be further compared with the obtained parameters from both tensile loading and longitudinal wave, with the deviation percentage listed in Table 6. It is concluded that

(1) The deviation percentage of $C_{11}$ reaches 17.80%, which indicates that the derived $C_{11}$ from tensile loading is slightly smaller than the value from GW testing. Considering the GW-based GA obtains three values of $C_{11}$ with only a maximum deviation of 1.42%, the tensile loading may not accurately reconstruct the value of $C_{11}$, possibly attributed to the incorrect cutting of the sample for tensile loading, which is further discussed in Section 3.3.1.
(2) The deviation percentage of $C_{12}$ reaches 61.79%, which can be attributed to the insensitiveness of GW on the change of $C_{12}$. This implies that more GW modes at a wider frequency range should be considered to accurately reconstruct the value of $C_{12}$.
(3) The deviation percentage of $C_{22}$ reaches 14.98%, indicating a good match. The one from the longitudinal wave actually measures the stiffness along the thickness direction, while the one from the GW combines the stiffness from both the transverse and thickness directions. This implies that the assumption of transverse isotropy is approximated and satisfied.
(4) The deviation percentage of $C_{23}$ reaches 10.82%, indicating a good match.
(5) The deviation percentage of $C_{66}$ reaches 0.53%, indicating an almost exact match.

In conclusion, excluding $C_{12},$ which exerts a negligible influence on GW dispersion, an average deviation of 11.03% for the four remaining parameters in UD is achieved between the GW-based GA and the technique combining static tensile loading and longitudinal wave testing. To achieve an accurate reconstruction of $C_{12}$, more GW modes in a wider frequency band should be involved, as discussed in Section 3.3.2. 

### 3.3. Further Refinement of Stiffness Matrix

#### 3.3.1. Refinement of $C_{11}$

**Figure 10:**
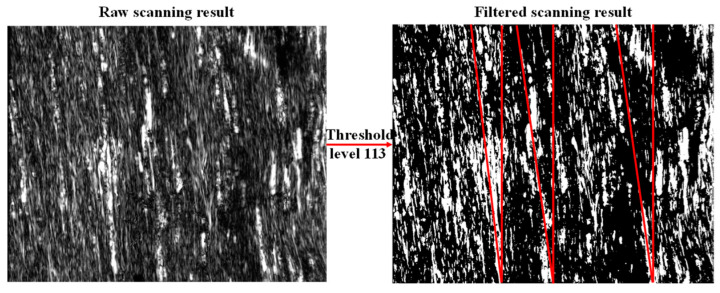
Ultrasound C-scan imaging of UD.

The reconstructed values of $C_{11}$ are close to each other among the CFRPs with three stacking sequences while different from the value measured with the tensile loading test. Thus, an ultrasound C-scan of UD used for tensile loading was performed with the ultrasonic microscope (PVA TePla^®^ SAM 401) to obtain raw imaging, which was further filtered with a threshold level of 113 for the measurement of fiber orientation with respect to the long edge of the sample, as displayed in Figure 10. An average angle of 9.67° was obtained with three measurements. To validate the influence of the incorrect cutting on the material parameter, a finite element modeling was performed with the material parameter listed in the second line of Table 5. After a rotation of material orientation of 9.67°, the calculated $E\prime_{1}$ along the long edge was 102.3 GPa, close to the calculated $E_{1}$ with only a slight deviation of 2.78 GPa, or a percentage of 2.72%. Hence, it was proved that the incorrect cutting of the sample for tensile loading resulted in a large deviation of $E_{1}$, which dominantly determined the value of $C_{11}$. Also, this implies that the GW-based GA reconstructed the value of $C_{11}$ with only a deviation percentage of around 2.72% to the tensile loading-based standard method.

#### 3.3.2. Refinement of $C_{12}$

**Figure 11:**
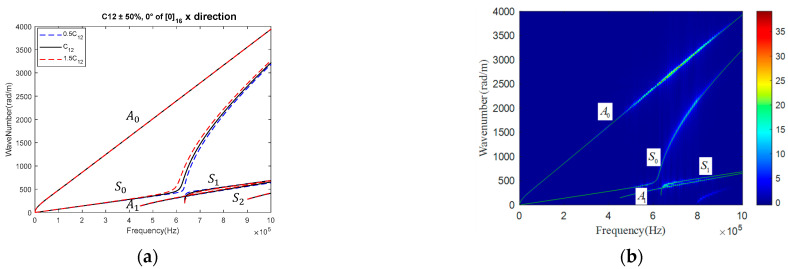
(**a**) Dispersion curve of GW along the fiber direction in UD, and (**b**) contour of frequency-wave number spectrogram superposed with the dispersion curve of GW based on the reconstructed $C_{12}$.

As $C_{12}$ is almost insensitive to the GW within the frequency range [0, 500 kHz] along both the x and z directions of CFRPs with three different stacking sequences, more GW modes within a broader frequency range [0, 1000 kHz] along the fiber direction in UD were investigated. The thickness was set at 2.05 mm, and material parameters were set according to the second line of Table 5, except that $C_{12}$ was taken at 5.549 GPa. Keeping other parameters unchanged and $C_{12}$ changed by 50%, the obtained GW dispersion curve is shown in Figure 11a. It was shown that the $S_{0}$ mode showed a moderate sensitivity to $C_{12}$. Thus, a time domain finite element analysis was performed with Abaqus/Explicit. Element CPE4R of size 0.15 mm was meshed, and a 3-cycle sinusoidal tone burst with a central frequency of 650 kHz was excited to acquire the response at 467 consecutive points at an interval of 0.15 mm. Following the same signal processing method with a 2-D FFT, $C_{12}$ was set as the only variable in the GA. The final reconstructed value $C_{12}$ was 5.333 GPa, which was with a deviation of 0.216 GPa, or a percentage of 3.9%, to the value calculated with tensile loading and longitudinal wave. Thus, it is expected that with multiple modes of GW in a wide frequency band, all the parameters in the stiffness matrix can be accurately characterized.

.

## 4. Conclusions

Multiple testing methods were performed, including GW, static tensile loading, and normally incident longitudinal waves, to reconstruct the five independent parameters in the stiffness matrix of CFRPs with unidirectional ([0/16]_s_), cross-ply ([0/90]_4s_), and quasi-isotropic ([0/90/0/90/45/–45/45/–45]_s_) stacking sequences. Compared with the destructive tensile loading-based technique, the proposed GW-based GA realized an automatic and nondestructive reconstruction of the stiffness matrix. For the four independent parameters in each ply of the laminate, excluding $C_{12}$, an average value of the maximum deviation of 11.62% was achieved among the CFRP with three different stacking sequences, and an average deviation of 11.03% for UD was achieved for the parameters measured based on different methods. Toward a further refinement of parameter characterization, the incorrect cutting of UD was compensated to reach a relative deviation of 2.72% for $E_{1}$ between a GW-based GA and destructive tensile loading. Also, with a finite element simulation that involved GW in a wider frequency range, it was proved that $C_{12}$ can also be reconstructed with a relative deviation of 3.9%. Future work will explore the excitation and acquisition of GWs experimentally up to a wider frequency range in order to realize the simultaneous and more accurate reconstruction of a stiffness matrix. In addition, a GA usually takes around 20 min to reconstruct the parameters, which is far from the realization of real-time parameter reconstruction. Hence, the improvement of reconstruction efficiency will be focused on. Finally, an extension of the proposed technique to various materials will also be investigated in the near future.

## Figures and Tables

**Table 1:** Parameters of CFRP samples, load, and sensor for tensile loading.

Cutting Direction	Size (mm)	Maximum Load (kN)	Resistance of Strain Gauge (Ω)
0° 45° 90°	250 × 25	2 for 0° 1.5 for 45° 1 for 90°	350

**Table 2:** CFRP sample specification.

No.	Stacking Sequence	Thickness (mm)	Density (kg/m^3^)
1	[0]_16_	2.05	1507.2
2	[0/90]_4s_	2.17	1490.5
3	[0/90/0/90/45/–45/45/–45]_s_	2.27	1480.1

**Table 3:** Parameters of GW testing.

CFRP Size (mm)	Piezoelectric Wafer	Excitation Central Frequency (kHz)	Array Spacing (mm)	Point Number
500 × 500	Φ8, 0.48 thick	400	1	128

**Table 4:** Derived stiffness matrix of UD with tensile loading and longitudinal wave.

$E_{1}$	$E_{3}$	$v_{13}$	$G_{13}$	$v_{23}$
99.55 GPa	9.305 GPa	0.3231	4.009 GPa	0.4389
$C_{11}$	$C_{12}$	$C_{22}$	$C_{23}$	$C_{66}$
103.1 GPa	5.549 GPa	11.82 GPa	5.352 GPa	4.009 GPa

**Table 5:** Reconstructed stiffness matrix and maximum deviation percentage.

	Ply Stacking	Resolution	$C_{11}$	$C_{12}$	$C_{22}$	$C_{23}$	$C_{66}$
Value (GPa)	UD	251 × 251	121.10	2.08	9.33	3.67	4.23
		501 × 501	125.42	3.43	10.28	4.83	4.03
		1001 × 1001	129.79	2.87	8.94	3.22	4.13
		2001 × 2001	110.50	6.13	9.96	4.56	4.23
	CP	251 × 251	129.86	5.41	8.54	2.06	3.16
		501 × 501	128.70	4.69	9.57	3.67	3.55
		1001 × 1001	129.18	6.05	9.96	3.56	3.26
		2001 × 2001	126.78	5.73	11.54	4.94	3.16
	QI	251 × 251	125.42	2.63	11.30	3.89	3.06
		501 × 501	126.58	3.90	10.75	3.22	3.06
		1001 × 1001	128.63	2.63	9.33	3.67	4.22
		2001 × 2001	127.95	4.22	12.32	4.83	3.06
Maximumdeviation (%)		251 × 251	3.50	60.38	16.21	35.76	21.44
		501 × 501	1.42	17.05	7.80	23.63	13.63
		1001 × 1001	2.93	21.82	14.24	38.66	20.93
		2001 × 2001	2.67	26.11	9.79	40.75	15.69

**Table 6:** Deviation percentage of the measured stiffness matrix with the image resolution 501 × 501.

Value	$C_{11}$	$C_{12}$	$C_{22}$	$C_{23}$	$C_{66}$
(%)	17.80	61.79	14.98	10.82	0.53

## Data Availability

Not applicable.

## Nomenclature

CFRP	carbon fiber reinforced polymer
GA	genetic algorithm
GW	guided wave
SAFE	semi-analytical finite element method
2D-FFT	two-dimensional Fourier transform
OF	objective function
$\sigma_{i}$	stress
$\varepsilon_{i}$	strain
$C_{ij}$	elastic constants
$E_{i}$	Young’s modulus
$v_{ij}$	Poisson’s ratio
$G_{ij}$	shear modulus
$N_{i}$	loading force
A	cross-sectional area of tensile sample
d	the measured thickness of tensile sample
$M_{cal}$	theoretically calculated images
$M_{\exp}$	experimental images
UD	unidirectional laminate [0]_16_
CP	cross-ply laminate [0/90]_4s_
QI	quasi-isotropic laminate [0/90/0/90/45/–45/45/–45]_s_
